# TDP-43-regulated cryptic RNAs accumulate in Alzheimer’s disease brains

**DOI:** 10.1186/s13024-023-00646-z

**Published:** 2023-08-21

**Authors:** Virginia Estades Ayuso, Sarah Pickles, Tiffany Todd, Mei Yue, Karen Jansen-West, Yuping Song, Jesús González Bejarano, Bailey Rawlinson, Michael DeTure, Neill R. Graff-Radford, Bradley F. Boeve, David S. Knopman, Ronald C. Petersen, Dennis W. Dickson, Keith A. Josephs, Leonard Petrucelli, Mercedes Prudencio

**Affiliations:** 1 Department of Neuroscience, Mayo Clinic, Jacksonville, FL USA; 2 Neuroscience Graduate Program, Mayo Clinic Graduate School of Biomedical Sciences, Jacksonville, FL USA; 3 Department of Neurology, Mayo Clinic, Jacksonville, FL USA; 4 Department of Neurology, Mayo Clinic, Rochester, MN USA; 5 Department of Research, Neuroscience, Mayo Clinic College of Medicine, 4500 San Pablo Rd, Jacksonville, FL 32224 USA

**Keywords:** TDP-43, Alzheimer’s disease, Cryptic RNA, LATE, STMN2

## Abstract

**Background:**

Inclusions of TAR DNA-binding protein 43 kDa (TDP-43) has been designated limbic-predominant, age-related TDP-43 encephalopathy (LATE), with or without co-occurrence of Alzheimer’s disease (AD). Approximately, 30–70% AD cases present TDP-43 proteinopathy (AD-TDP), and a greater disease severity compared to AD patients without TDP-43 pathology. However, it remains unclear to what extent TDP-43 dysfunction is involved in AD pathogenesis.

**Methods:**

To investigate whether TDP-43 dysfunction is a prominent feature in AD-TDP cases, we evaluated whether non-conserved cryptic exons, which serve as a marker of TDP-43 dysfunction in amyotrophic lateral sclerosis (ALS) and frontotemporal lobar degeneration (FTLD-TDP), accumulate in AD-TDP brains. We assessed a cohort of 192 post-mortem brains from three different brain regions: amygdala, hippocampus, and frontal cortex. Following RNA and protein extraction, qRT-PCR and immunoassays were performed to quantify the accumulation of cryptic RNA targets and phosphorylated TDP-43 pathology, respectively.

**Results:**

We detected the accumulation of misspliced cryptic or skiptic RNAs of *STMN2*, *KCNQ2*, *UNC13A*, *CAMK2B*, and *SYT7* in the amygdala and hippocampus of AD-TDP cases. The topographic distribution of cryptic RNA accumulation mimicked that of phosphorylated TDP-43, regardless of TDP-43 subtype classification. Further, cryptic RNAs efficiently discriminated AD-TDP cases from controls.

**Conclusions:**

Overall, our results indicate that cryptic RNAs may represent an intriguing new therapeutic and diagnostic target in AD, and that methods aimed at detecting and measuring these species in patient biofluids could be used as a reliable tool to assess TDP-43 pathology in AD. Our work also raises the possibility that TDP-43 dysfunction and related changes in cryptic splicing could represent a common molecular mechanism shared between AD-TDP and FTLD-TDP.

**Supplementary Information:**

The online version contains supplementary material available at 10.1186/s13024-023-00646-z.

## Background

Alzheimer’s disease (AD) is the most common cause of dementia worldwide, displaying an insidious onset with a gradual progression of memory loss and cognitive decline. The neuropathological mechanisms of AD are related to abnormalities in the proteins β-amyloid and tau, which aggregate in neuritic plaques and neurofibrillary tangles (NFTs), respectively [1]. Approximately, 30–70% of AD patients are also affected by TAR DNA-binding protein 43 kDa (TDP-43) proteinopathy [2–8]. In fact, TDP-43 pathology can be found in aging brains, which has been termed limbic-predominant, age-related TDP-43 encephalopathy (LATE), and may be concomitant with AD neuropathological changes [9–11]. TDP-43 is an RNA-binding protein strongly linked to the majority of cases of amyotrophic lateral sclerosis (ALS) and about 50% of cases of frontotemporal lobar degeneration (FTLD-TDP) [12, 13]. AD cases affected by TDP-43 proteinopathy (AD-TDP) present with a greater disease severity characterized by worse memory and greater hippocampal atrophy compared to AD patients without TDP-43 pathology [14–16]. Currently, there is a lack of understanding of the mechanism/s underlying TDP-43-associated neurodegeneration in AD pathogenesis.

TDP-43 is a nuclear protein with roles in maintaining RNA homeostasis [17–19]. In TDP-43 proteinopathies, TDP-43 becomes insoluble and either aggregates in the nucleus or mislocalizes to the cytoplasm and forms inclusions there – ultimately leading to a loss of its nuclear function [12, 13, 20]. The molecular pattern of TDP-43 deposition has been used to establish a staging scheme for AD-TDP [21–23]: the amygdala becomes affected in the first stages followed by progression into hippocampal regions. In the most severe and advanced cases, TDP-43 inclusions can also be detected in the frontal cortex. In contrast, in FTLD-TDP cases, TDP-43 positive inclusions are initially detected in the amygdala, but progress through the medial frontal cortex, hippocampus and, in latter stages, spread toward motor cortex, spinal cord, and occipital lobe [24]. Further, TDP-43 histopathological pattern deposition is heterogeneous, and has been used to classify TDP-43 cases into different subtypes. In approximately 54% of AD-TDP cases, TDP-43 inclusions resemble the aggregates described in FTLD-TDP type A cases, which are characterized by neuronal cytoplasmic and intranuclear inclusions, dystrophic neurites, and are more widely distributed (AD-TDP type α) [25, 26]. In the remaining 46% of AD-TDP cases, TDP-43 inclusions are accompanied by TDP-43 associated with NFTs, which has been referred to as AD-TDP type β [25, 26]. Nevertheless, although AD-TDP and FTLD-TDP are classified by distinct neuropathological and clinical presentations, TDP-43 dysfunction in both disorders suggests they have a common molecular mechanism.

The role of TDP-43 as a splicing repressor has recently received increased attention in light of the discovery that loss of TDP-43 function leads to inclusion of non-conserved cryptic exons in ALS/FTLD-TDP and AD cases [19, 27–34]. The two most studied cryptic RNAs are the cryptic transcript variants of stathmin-2 (*STMN2*) and unc-13 homolog A (*UNC13A*). STMN2 is a microtubule-associated phosphoprotein involved in signal transduction and neuronal growth [31, 32, 35], and with roles in maintaining neuromuscular junctions [36–39]. Whereas *UNC13A*, a gene harboring ALS and FTD-linked risk variants, encodes a protein involved in neurotransmitter release [29, 30]. Both cryptic species accumulate in the frontal cortex of FTLD-TDP cases, and associate with the burden of phosphorylated TDP-43 (pTDP-43). Further, they are also associated with an earlier age of disease onset and a shorter survival [29, 35]. The fate of different cryptic RNAs varies: some species, including the cryptic forms of *STMN2* and *UNC13A*, incorporate premature stop codons resulting in their rapid degradation without undergoing translation. In contrast, other cryptic RNAs are effectively translated, and novel peptides have been detected in neurons and cerebrospinal fluid (CSF) of ALS with or without frontotemporal dementia (FTD) patients [34, 40], potentially serving as markers of TDP-43 dysfunction. Regardless whether the cryptic RNA may lead to a truncated variant or not, the accumulation of cryptic RNAs results in downregulation of the corresponding full-length RNA and protein variants, many of which have important roles in maintaining neuronal functions [29–32, 35, 41]. Rescuing these missplicing events may present a novel approach to mitigate TDP-43-related neurotoxicity [32, 42]. Thus, cryptic RNA accumulation due to TDP-43 dysfunction may have potential not only as a biomarker to stratify patients with and without TDP-43 pathology, but may also have important therapeutic implications for TDP-43 proteinopathies.

Although aberrant cryptic transcripts have been systematically reported in ALS/FTLD-TDP cases, the degree to which these RNAs accumulate in other TDP-43 proteinopathies such as AD, was unknown. Therefore, we aimed to investigate if cryptic RNAs detected in FTLD-TDP also accumulate in AD-TDP in three brain regions differentially affected by TDP-43 deposition (amygdala, hippocampus, and frontal cortex) [21–23]. Compared to cognitively normal controls and AD cases without TDP-43 pathology, AD-TDP cases accumulated cryptic RNAs in regions most affected by TDP-43 deposition. Similar to what has been observed for FTLD-TDP, some cryptic RNAs in AD-TDP best associated with the levels of pTDP-43 and discriminated from TDP-43 negative controls. These results suggest that different TDP-43 proteinopathies may share common pathological mechanisms, and TDP-43-targeted interventions and associated diagnostic tools may be of benefit to multiple conditions, including AD-TDP as well as FTLD-TDP.

## Methods

### Study approval and sample selection

Human postmortem brain tissues from amygdala, hippocampus, and frontal cortex were provided by the Mayo Clinic Florida Brain Bank. Diagnosis was independently ascertained by trained neurologists and neuropathologists upon neurological and pathological examinations, respectively. All participants, or the next of kin, provided written informed consent, and all protocols were reviewed and approved by the Mayo Clinic Institutional Review Board and Ethics Committee. Samples were selected based on neuropathological diagnosis of FTLD-TDP, AD with and without TDP-43 pathology, and cognitively normal controls. Of note, the presence/absence of Lewy bodies was not a criterion for sample selection. A total of 15 AD-TDP cases (21%) had Lewy bodies in the amygdala, but none had Lewy bodies in the brainstem or cortex. Size of the study cohort was determined by sample availability for all three brain regions, TDP-43 subtype characterization available, and both males and females were included in the study. A summary of the study cohort is described in Table 1.

**Table 1:** Study cohort characteristics

Study group	N	Age of death (yrs)	N females (%)	Braak	Thal	Duration (yrs)	Age of onset (yrs)
Cognitively normal (CN)	27	79 (54, 95)	10 (37%)	II (0, IV)	0 (0, 3)	NA	NA
AD no TDP	27	78 (59, 89)	11 (40.7%)	VI (V, VI)	5 (4, 5)	9 (1, 14)	70 (54, 84)
AD-TDP	71	84 (62, 101)	47 (66.2%)	VI (IV, VI)	5 (3, 5)	10 (3, 23)	73 (51, 94)
AD TDP type α	36	86 (68, 101)	21 (58.3%)	VI (IV-V, VI)	5 (3, 5)	9 (3, 18)	73 (60, 94)
AD TDP type β	35	83 (62, 92)	26 (76.5%)	VI (IV, VI)	5 (3, 5)	10.5 (3, 23)	72 (51, 87)
FTLD-TDP	67	71 (45, 91)	32 (47.8%)	I-II (0, IV-V)	1 (0, 5)	5 (1, 25)	64 (42, 90)
FTLD-TDP type A	32	77 (51, 91)	16 (50%)	I (0, III-IV)	1 (0, 5)	6 (1, 25)	68 (47, 90)
FTLD-TDP type B	35	72 (45, 87)	16 (45.7%)	I-II (0, IV-V)	0.5 (0, 5)	4 (1, 20)	63 (42, 82)

yrs: years. The sample median (minimum, maximum) is given for continuous variables (age, disease duration, Braak, Thal), categorical variables (sex) were summarized with number and percentage of patients. Information was unavailable regarding age at disease onset and disease duration for 4 AD no TDP, 2 AD-TDP type α, and 2 (onset) – 3 (duration) AD-TDP type β; age at death for 1 AD-TDP type β. NA: not applicable.

### Neuropathological assessments

Immunohistochemistry was performed on 5-µm-thick sections of formalin-fixed, paraffin-embedded tissue mounted on glass slides. Sections were deparaffinized and processed for phospho-TDP-43 (pS409/410, mouse monoclonal, 1:5,000; Cosmo Bio, Tokyo, Japan) [25], and Thioflavin S (Sigma-Aldrich, St. Louis, MO) for Braak staging [43] and Thal phasing [44], according to previously published methods.

### Tissue sampling for biochemical assessments

In order to determine pTDP-43 protein and cryptic RNA levels, 50-60 mg of human tissue for protein and 40 mg tissue for RNA extractions were dissected from the amygdala, hippocampus and frontal cortex. Specifically, the amygdala was cut from a coronal section at the level of the uncus, the hippocampus was cut from a coronal section at the level of the lateral geniculate, and the middle frontal gyrus samples were cut from Brodmann area 9.

### Protein extraction from human brain tissues

To measure pTDP-43 from postmortem tissues, we performed protein fractionation as previously described [45]. Briefly, tissues were homogenized in 5 volumes (w/v) of cold RIPA buffer (25 mM Tris-HCl [pH 7.6], 150 mM NaCl, 1% sodium deoxycholate, 1% Nonidet P-40, 0.1% sodium dodecyl sulfate, and protease and phosphatase inhibitor cocktail), sonicated on ice, and centrifuged at 100,000 x g for 30 min at 4 °C. The supernatant was then collected as RIPA-soluble fraction. The pellet was resuspended in RIPA buffer, sonicated and centrifuged again. The supernatant was discarded, and the remaining pellet was dissolved in urea buffer (30 mM Tris-HCl [pH 8.5], 7 M urea, 2 M thiourea, and 4% CHAPS [(3-[(3-cholamidopropyl)dimethylammonio]-1-propanesulfonate]) for 1 h at room temperature with continuous agitation. Following incubation, samples were sonicated and centrifuged at 100,000 x g for 30 min at 22 °C. The resulting supernatant, referred as urea-soluble or RIPA-insoluble fraction, was then collected. Protein concentrations of urea-soluble fractions were determined by Bradford assay (ThermoFisher).

### Phosphorylated TDP-43 immunoassay

Assessment of pTDP-43 was performed in the urea-soluble fraction from the amygdala, hippocampus, and frontal cortex of our study cohort using a sandwich Meso Scale Discovery (MSD) immunoassay [45]. A rabbit polyclonal antibody specific for TDP-43 phosphorylated at serines 409/410 (3 µg/mL, 22309-1-AP, Proteintech) was used for capture, and a sulfo-tagged rabbit polyclonal C-terminal TDP-43 antibody (3 µg/mL, 12892-1-AP, Proteintech) for detection. All samples were tested in duplicate wells, and controls were included in every plate to account for any interplate variability. MSD QUICKPLEX SQ120 technology was used to acquire the response values corresponding to the intensity of emitted light upon electrochemical stimulation.

### RNA extraction from human brain samples

RNA was extracted using the RNAeasy Plus Mini Kit (Qiagen) per manufacturer’s instructions, and as previously described [35, 45]. RNA from up to three cuts was extracted, and only extractions with high quality RNA were kept for downstream analyses. RNA concentration was determined using Nanodrop technologies (Thermo Fisher), and an Agilent 2100 bioanalyzer (Agilent Technologies) was used to evaluate the RNA integrity number (RIN). Median RIN values for all samples were > 9 for all brain regions (Table S1).

### Evaluation of cryptic RNA accumulation by qRT-PCR

Following RNA extraction, 500 ng of total RNA were transcribed into complementary DNA (cDNA) using the High-Capacity cDNA Transcription Kit (Applied Biosystems) according to manufacturer’s directions. Then, quantitative real-time PCR (qRT-PCR) was performed, in triplicates, using SYBR GreenER qPCR SuperMix (Invitrogen) on a QuantStudio™ 7 Flex Real-Time PCR System (Applied Biosystems). All samples from amygdala, hippocampus and frontal cortex were assessed at the same time, and control samples were included in every plate to account for any interplate variability. Relative quantification of cryptic RNAs was determined using the ΔΔCt method and normalized to two endogenous controls, *GAPDH* and *RPLP0.* The following primers were used: cryptic *STMN2* forward: 5′-GGACTCGGCAGAAGACCTTC-3′, cryptic *STMN2* reverse: 5′-GCAGGCTGTCTGTCTCTCTC-3′; skiptic *KCNQ2* forward: 5′-TATGCCCACAGCAAGATCAC-3′, skiptic *KCNQ2* reverse: 5′-AGACACCGATGAGGGTGAAG; cryptic *UNC13A* forward: 5′-TGGATGGAGAGATGGAACCT, cryptic *UNC13A* reverse: 5′-GGGCTGTCTCATCGTAGTAAAC; cryptic *CAMK2B* forward: 5′-CTGCTCCGTGGTCTTAATGAT, cryptic *CAMK2B* reverse: 5′-GAGTGCAGAGACTTCCCCC; cryptic *SYT7* forward: 5′-GCAGTGAGAAGAAGGCTATCAA; cryptic *SYT7* reverse 5′-CGGCAGACTGGAGCCT; *GAPDH* forward: 5′-GTTCGACAGTCAGCCGCATC, *GAPDH* reverse: 5′-GGAATTTGCCATGGGTGGA; and *RPLP0* forward: 5′-TCTACAACCCTGAAGTGCTTGAT; *RPLP0* reverse: 5′-CAATCTGCAGACAGACACTGG.

### Statistics

Statistical analyses were performed in GraphPad Prism 9 (GraphPad Software). To evaluate differences in pTDP-43 protein or cryptic RNA levels between disease cases and controls, for each region, we performed One-way ANOVA followed by Dunn’s multiple comparison tests as indicated in the figure legends. When comparing cryptic RNA levels between AD-TDP to AD no TDP cases, the Mann-Whitney test was used. Further, we also performed single-variable (unadjusted) and multivariable linear regression models (adjusted) to evaluate differences between AD-TDP and other study groups (Tables). Multivariable models were adjusted for age at death, sex and RIN for cryptic RNAs, and for age at death and sex for pTDP-43 protein. Due to the skewed distribution of the data, cryptic RNA and pTDP-43 were analyzed on the base 10 logarithmic scale. The regression coefficients (β) and 95% confidence intervals (CIs) were estimated and interpreted as the difference in the means, bases on the 10 logarithmic scale, between all AD-TDP cases (reference group) and the other study groups. For pTDP-43, *P* values less than 0.0167 were considered statistically significant after adjusting for the three separate AD-TDP vs. CN, AD-TDP vs. AD no TDP, and AD-TDP vs. FTLD-TDP analyses. For cryptic RNA, *P* values less than 0.025 were considered statistically significant after adjusting for the three separate AD-TDP vs. controls (CN + AD no TDP), and AD-TDP vs. FTLD-TDP analyses.

Associations between cryptic RNA and pTDP-43 protein levels were evaluated in AD-TDP and FTLD-TDP cases using single-variable and multivariable linear regression models. Both cryptic RNA and pTDP-43 protein levels were analyzed using the base 10 logarithmic scale. The multivariable model was adjusted for age, sex, RIN, and TDP-43 subtype, and *P* values < 0.01 were considered as statistically significant. For assessing differences in cryptic RNA levels within TDP-43 subtypes, in AD-TDP and FTLD-TDP, single-variable and multivariable linear regression models were also performed as described, and where TDP-43 type A/α was set as the reference group. The multivariable model was adjusted for age, sex, RIN, and pTDP-43 levels, and P values < 0.05 were considered as statistically significant.

To evaluate the ability of cryptic RNAs to discriminate AD-TDP or FTLD-TDP cases from controls, we estimated the area under the receiver operating characteristic curve (AUC) along with a 95% confidence interval (CI) for each of the cryptic RNAs in each region. Of note, an AUC value of 0.5 corresponds to predictive ability equal to that of chance, and an AUC of 1.0 represents perfect predictive ability.

## Results

### Study cohort characteristics

Our study cohort included a total of 192 postmortem cases classified into four main groups: 27 cognitively normal (CN) cases (median Braak NFT stage II, Thal phase 0), 27 AD cases without TDP-43 pathology (AD no TDP), 71 AD cases with confirmed TDP-43 pathology (AD-TDP), and 67 FTLD-TDP cases (Table 1). All AD cases had a median Braak NFT stage of VI and Thal phase of 5, while FTLD-TDP cases presented lower Braak NFT (median I-II, range: 0 to IV-V) and Thal phase (median 1, range: 0–5) (Table 1). The median age of death for the AD-TDP group was 84 years, the FTLD-TDP median age of death was lower at 71 years, and the median age of death of CN and AD no TDP-43 were 79 and 78 years, respectively (Table 1). Our study included similar numbers of males and females, when possible, based on tissue availability. The AD-TDP and CN groups showed the highest and lowest proportion of females, respectively (Table 1). Disease duration in AD cases, irrespective of the presence of TDP-43 pathology, was 9–10 years and with a median age of disease onset was 70–73 years (Table 1). As expected, disease duration in FTLD-TDP cases was shorter (median 5 years, range: 1–25 years) and disease onset was earlier (median 64 years, range: 42–90 years) (Table 1). Both, AD-TDP and FTLD-TDP groups included similar number of cases from two TDP-43 subtypes: type α (N = 36) and β (N = 35) in AD-TDP, and type A (N = 32) and B (N = 35) in FTLD-TDP (Table 1).

### TDP-43 pathology accumulates in amygdala and hippocampus of AD-TDP

**Fig. 1:**
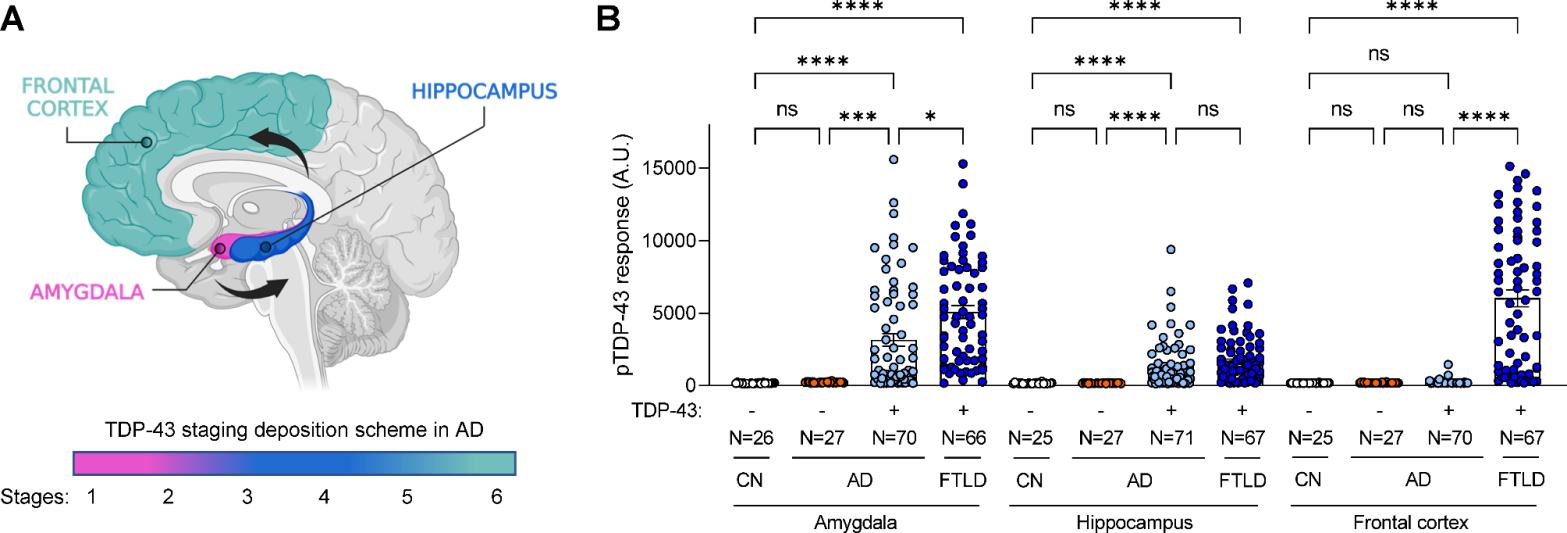
TDP-43 pathology accumulates in amygdala and hippocampus of AD-TDP. (**A**) Graphical overview of AD-TDP staging based on TDP-43 deposition pattern from amygdala (stage 1), followed by the hippocampus and occipitotemporal gyrus (stages 2–3), basal forebrain and ventral striatum (stages 4–5) and last in the frontal cortex (stage 6). Created with BioRender.com. (**B**) Quantification of pTDP-43 protein levels in cognitively normal controls (CN), AD and FTLD cohorts across three brain regions: amygdala, hippocampus, and frontal cortex (see Table 1), using an immunoassay (see **Methods**). Data are presented as mean ± SEM. Number of cases is included in the figures. Statistical analyses were performed by One-way ANOVA following Dunn’s multiple comparison tests: *P < 0.05, **P < 0.005, *** P < 0.0005, ****P < 0.0001, ns: not significant

To assess TDP-43 dysfunction in AD-TDP, we first evaluated post-mortem brain tissues from three different brain regions: amygdala, hippocampus, and frontal cortex. The criteria for selecting these specific areas were based on TDP-43 deposition scheme in AD that describes an initial TDP-43 accumulation in the amygdala (stage 1) followed by pathology in hippocampus and occipitotemporal gyrus (stages 2–3), basal forebrain and ventral striatum (stages 4–5) and last in the frontal cortex (stage 6) (Fig. 1A) [21–23]. Importantly, these same regions are known to accumulate TDP-43 pathology in FTLD-TDP [24, 46], allowing us to evaluate whether changes resulting from TDP-43 dysfunction in FTLD-TDP are also observed in AD-TDP. Given that pTDP-43 is a sensitive and specific marker of TDP-43 pathology [29, 35, 45], we quantified pTDP-43 protein levels across all three brain regions using a Meso Scale Discovery (MSD) based immunoassay. We found a significant accumulation of pTDP-43 protein in the urea soluble fraction of all three regions (amygdala, hippocampus, and frontal cortex) of FTLD-TDP cases (Fig. 1B). In AD-TDP, pTDP-43 protein levels were markedly elevated in the amygdala, the primarily region affected by TDP-43 pathology; levels were also significantly elevated in the hippocampus, albeit to a lesser extent (Fig. 1B). No significant pTDP-43 accumulation was found in the frontal cortex of AD-TDP cases (Fig. 1B), consistent with AD-TDP pathophysiology where a very small proportion of cases present TDP-43 pathology in this region [21–23]. As expected, all control cases, regardless of their classification as cognitively normal (CN) or AD without TDP-43 (AD no TDP), showed no pTDP-43 accumulation in any region (Fig. 1B). Further, the accumulation of pTDP-43 in the amygdala and hippocampus of AD-TDP cases was significantly increased compared to controls (CN and AD no TDP), and significantly lower than FTLD-TDP cases after adjusting for sex, and age at death (Table S2).

### Aberrant cryptic RNAs accumulate in the amygdala and hippocampus of AD-TDP

**Fig. 2:**
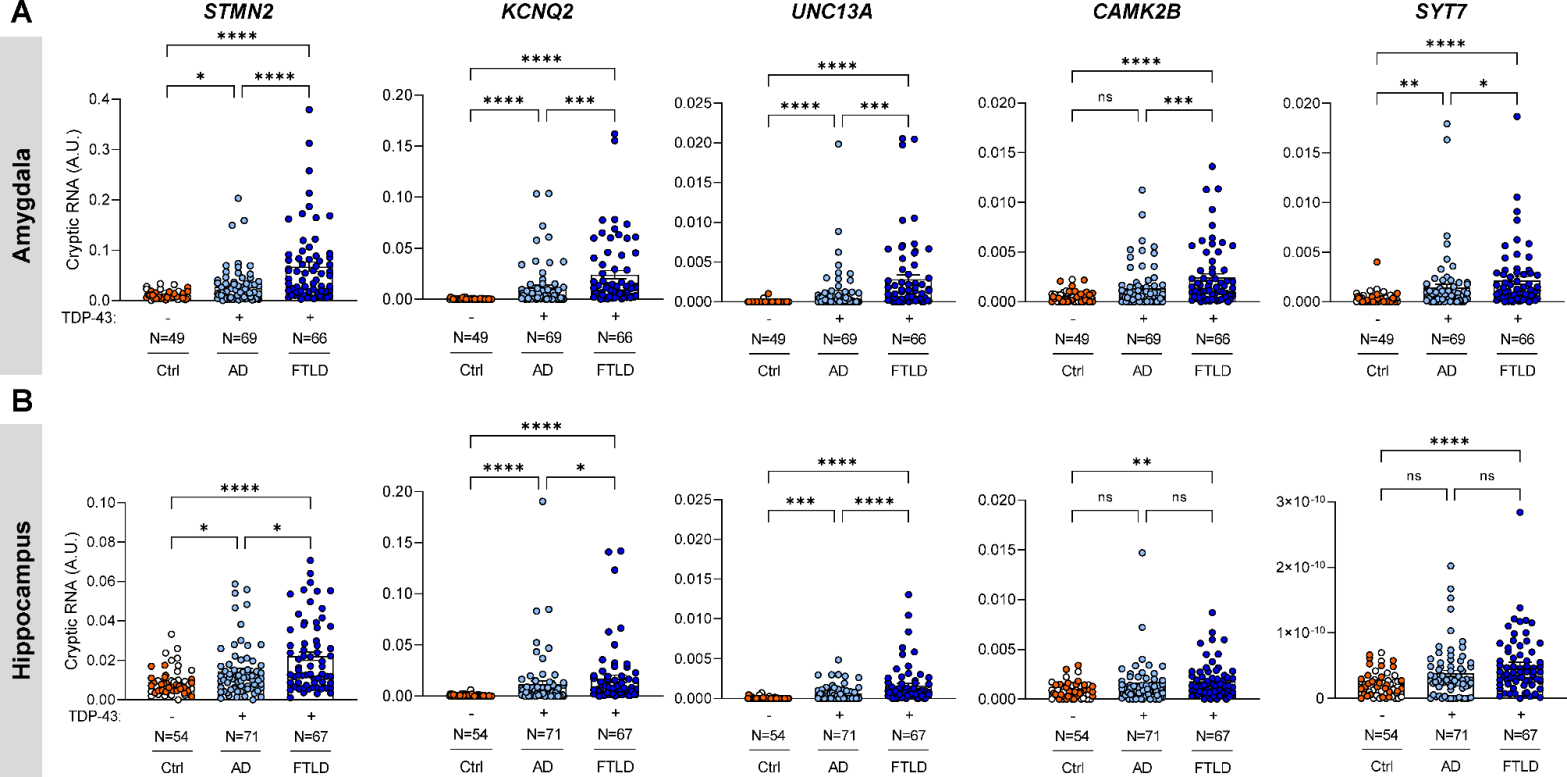
Aberrant cryptic RNAs accumulate in the amygdala and hippocampus of AD-TDP. Cryptic RNA (*STMN2, KCNQ2, UNC13A, CAMK2B, and SYT7*) levels were measured by qRT-PCR in amygdala (**A**) and hippocampus (**B**) of controls (Ctrl: CN, represented by white circles + AD no TDP, represented by orange circles), AD-TDP, and FTLD-TDP cases. Number of cases is included in the figures. Data are presented as mean ± SEM. Statistical analyses were performed by One-way ANOVA following Dunn’s multiple comparison tests: *P < 0.05, **P < 0.005, *** P < 0.0005, ****P < 0.0001, ns: not significant

Having validated the presence of TDP-43 pathology in different regions of AD-TDP and FTLD-TDP brains, we next sought to evaluate whether these same regions demonstrated evidence of TDP-43 dysfunction. The aberrant accumulation of cryptic RNA targets in ALS and FTLD tissues with TDP-43 pathology (e.g., frontal cortex, motor cortex, spinal cord, etc.) has been the focus of recent studies [29–32, 34, 35]. These studies found that, in the absence of TDP-43, cryptic RNAs are expressed from genes with key functions in neuronal networks. In particular, cryptic RNAs encoded from *STMN2* (neuronal growth, axonal regeneration, signal transduction), *KCNQ2* (neuronal excitability), *UNC13A* (neurotransmitter release at the synapse), *CAMK2B* (neuronal plasticity and synapse formation), and *SYT7* (exocytosis of secretory and synaptic vesicles) have been validated in frontal cortex of ALS and FTLD-TDP [29, 34, 35]. Moreover, some are detectable as peptides in ALS/FTD CSF [34]. In fact, we validated the accumulation of five cryptic RNAs in the frontal cortex of FTLD-TDP in this study cohort (Fig. S1, Table S3). Of note, we did not detect significant accumulation in the frontal cortex of AD-TDP cases, most likely due to the paucity of TDP-43 pathology in this region (see Fig. 1). In contrast, we found significant accumulations of cryptic RNAs in both amygdala (Fig. 2A) and hippocampus (Fig. 2B) of AD-TDP compared to controls (Ctrl: CN + AD no TDP), even after adjusting for potential confounding variables [age at death, sex and RNA integrity number (RIN), Table S4]. Of note, we combined CN and AD no TDP groups, referred as controls from herein, since both groups are negative for pTDP-43 accumulation. Nonetheless, similar results were found when comparing AD-TDP to AD no TDP (Fig. S2). Moreover, while the accumulation of cryptic RNAs appeared greater in FTLD-TDP, it was not significantly higher than in AD-TDP after adjusting for age at death, sex, and RIN (Table S4). Taken together, our results indicate that AD with TDP-43 pathology accumulate cryptic RNAs in brain regions affected by TDP-43 pathology.

**Table 2:** Cryptic RNA accumulation associates with higher pTDP-43 protein levels in AD-TDP.

	Unadjusted analysis		Adjusting for age at death, sex, RIN and TDP-43 type	
Group	Regression coefficient (95% CI)	P-value	Regression coefficient (95% CI)	P-value
**Amygdala**				
*STMN2*	0.007673 (0.002224 to 0.013120)	0.0065	0.009574 (0.003768 to 0.015380)	0.0016
*KCNQ2*	0.003975 (0.000553 to 0.007396)	0.0235	0.004944 (0.001248 to 0.008639)	0.0096
*UNC13A*	0.000310 (-0.000149 to 0.000769)	0.1819	0.000422 (-0.000203 to 0.001048)	0.0800
*CAMK2B*	0.000438 (8.75e-5 to 0.000789)	0.0151	0.000538 (0.000158 to 0.000918)	0.0620
*SYT7*	0.000137 (-0.000371 to 0.000645)	0.5368	0.000219 (-0.000337 to 0.000775)	0.4333
**Hippocampus**				
*STMN2*	0.002044 (-0.000590 to 0.004679)	0.1262	0.002504 (-0.000482 to 0.005490)	0.0988
*KCNQ2*	0.008518 (0.003578 to 0.013460)	0.0010	0.007834 (0.002402 to 0.013270)	0.0054
*UNC13A*	0.000259 (6.86e-5 to 0.000449)	0.0084	0.000313 (9.98e-5 to 0.000526)	0.0046
*CAMK2B*	3.50e-5 (-0.000383 to 0.000452)	0.8693	1.23e-5 (-0.000455 to 0.000480)	0.9583
*SYT7*	1.46e-11 (7.13e-12 to 2.12e-11)	0.0002	1.51e-11 (6.57e-12 to 2.37e-11)	0.0008

CI = confidence interval, RIN: RNA integrity number; regression coefficients, 95% CIs, and *P* values are shown for associations of pTDP-43 from unadjusted linear regression models or linear regression models adjusted for age, sex, RIN and TDP-43 subtype. *P* values < 0.01 are considered statistically significant.

### Cryptic RNA accumulation associates with pTDP-43 burden but does not generally differ between TDP-43 subtypes

In previous studies, we and others demonstrated that pTDP-43 burden is strongly associated with the levels of cryptic RNAs in the frontal cortex of ALS and FTLD-TDP. We also observed a correlation between pTDP-43 levels and the accumulation of all five cryptic RNAs in FTLD-TDP frontal cortex (Table S5). Interestingly, the correlation of pTDP-43 protein and cryptic RNAs was most significant in frontal cortex and amygdala, but less in the hippocampus (Table S5). In AD-TDP, we found significant associations between cryptic RNAs and pTDP-43 in both amygdala and hippocampus, even after adjusting for age at death, sex, RIN, and TDP-43 subtype (Table 2). Among all cryptic RNAs evaluated, *KCNQ2* cryptic RNA consistently associated with higher pTDP-43 burden in both brain regions (Table 2).

Next, we sought to evaluate whether the type and distribution of TDP-43 inclusions associated with the levels of cryptic RNAs. In FTLD-TDP, TDP-43 type A and B are the most frequent subtypes. TDP-43 type A has the most widely distributed TDP-43 pathology and presents a wider range of inclusion types, while TDP-43 type B is predominantly characterized by neuronal cytoplasmic inclusions and is sometimes associated with motor neuron disease [47]. In our study cohort, we observed that FTLD-TDP type B tended to have lower levels of pTDP-43 burden in the frontal cortex, although it did not reach statistical significance (Fig. S3A). Of all five cryptic RNAs, the levels of *KCNQ2* cryptic RNA in the frontal cortex and *CAMK2B* cryptic RNA in the amygdala were significantly lower in TDP-43 type B FTLD-TDP, after adjusting for age at death, sex, RIN and pTDP-43 levels (Table S6). No subtype-specific differences were observed for the other three cryptic RNAs (Table S6). In AD-TDP a similar TDP-43 staging scheme has also been described, with TDP-43 type α resembling TDP-43 type A in FTLD-TDP, and TDP-43 type β often associated with neurons that also have tau aggregates in the form of NFTs [25, 26]. Similar to what was observed in FTLD-TDP type B, AD-TDP cases with TDP-43 type β showed a lower accumulation of pTDP-43, but this was not significant (Fig. S3B). Only *STMN2* cryptic RNA levels in the amygdala were significantly lower in AD-TDP type β after adjusting for age at death, sex, RIN and pTDP-43 levels (Table S7). Once again, there was no change in the levels of the other cryptic RNAs across TDP-43 subtypes (Table S7). Overall, our results confirm strong associations of cryptic RNA accumulation with pTDP-43 burden in FTLD-TDP and AD-TDP. Moreover, our data demonstrated that cryptic RNA accumulation was largely independent of TDP-43 subtype.

### Cryptic RNAs can discriminate TDP-43 cases from controls

**Fig. 3:**
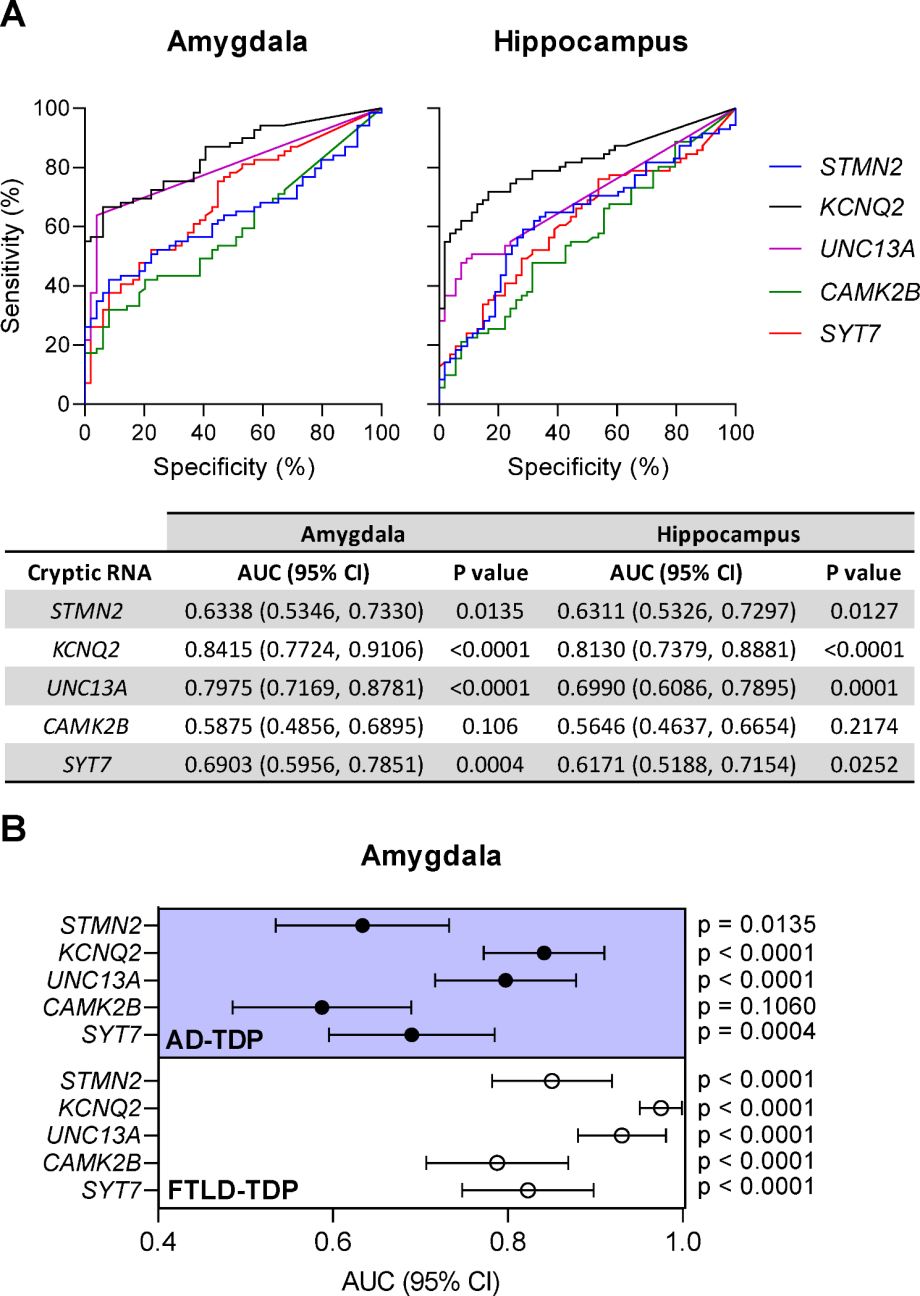
Cryptic RNA can discriminate AD-TDP cases from controls. (**A**) Representative images of the discriminatory ability of cryptic RNAs to distinguish AD-TDP cases from controls, evaluated by receiver operating characteristic (ROC) analyses, in amygdala (left; AD-TDP, N = 69; controls, N = 49) and hippocampus (right; AD-TDP, N = 71; controls, N = 54). The area under the curve (AUC) values, 95% confidence intervals (CI), and *P* values for each cryptic RNA are included in the bottom table. (**B**) Representative image of the comparable discriminatory ability pattern of cryptic RNAs for AD-TDP and FTLD-TDP cases in the amygdala

To evaluate the ability of each of the cryptic RNAs to distinguish TDP-43 positive from TDP-43 negative cases, we performed receiver operating characteristic (ROC) analyses and calculated the area under the curve (AUC) for each cryptic RNA in FTLD-TDP and AD-TDP compared to controls. As expected, the discriminatory ability of all five cryptic RNAs was highly significant (*P* < 0.0001) in the frontal cortex and amygdala in FTLD-TDP, with AUC ranging 0.79–0.98 in amygdala and 0.81–0.94 in frontal cortex (Fig. S4). The discriminatory ability of the cryptic RNAs in the hippocampus of FTLD-TDP cases was more variable (AUC: 0.67–0.95), but still statistically significant (*P* < 0.005, Fig. S4). In AD-TDP, four (*STMN2, KCNQ2, UNC13A, SYT7*) out of the five cryptic RNA were able to distinguish TDP-43 cases from controls, in both amygdala and hippocampus, although the significance and AUC values were lower in the latter region (Fig. 3A). Interestingly, *KCNQ2* and *UNC13A* cryptic RNA levels had the most significant discriminatory ability in AD-TDP. Of note, the pattern of discriminatory ability in AD-TDP was similar to that of FTLD-TDP, with *KCNQ2* and *UNC13A* cryptic RNAs showing the highest significance and AUC values (Fig. 3B). Overall, our data demonstrate that cryptic RNA levels display a reliable ability to discriminate TDP-43 positive cases from controls in both FTLD-TDP and AD-TDP.

## Discussion

TDP-43 dysfunction results in a lack of splicing repression leading to the aberrant accumulation of cryptic RNAs in ALS and FTLD-TDP brain regions with marked TDP-43 pathology [19, 27–35]. Here, we aimed to evaluate to what extent TDP-43 dysfunction is also present in AD-TDP by assessing accumulation of cryptic RNAs characteristic of FTLD-TDP in the amygdala, hippocampus and frontal cortex. Consistent with the topographic distribution of TDP-43 inclusions, pTDP-43 significantly accumulated in all three regions in FTLD-TDP, with amygdala and frontal cortex showing the highest burden. In contrast, AD-TDP had greater accumulation in the amygdala, followed by the hippocampus, and no detectable accumulation in frontal cortex. The latter is not surprising since only a small proportion of AD-TDP cases has TDP-43 pathology in the frontal cortex [21–23]. Interestingly, the levels of pTDP-43 in amygdala and hippocampus of AD-TDP were lower than those in FTLD-TDP, even after correcting for confounding variables. Given that the time course of FTLD-TDP is relatively shorter than that of AD-TDP, it is tempting to speculate that enhanced pTDP-43 deposition may correlate with the more aggressive disease course in FTLD-TDP. On the other hand, accumulation of pTDP-43 protein may represent only a fraction of reduced TDP-43 nuclear function. In fact, loss of nuclear TDP-43 without overt TDP-43 pathology has been associated with neurodegeneration [48, 49], and is sufficient to accumulate TDP-43 cryptic RNA targets [28, 41]. Thus, evaluating splicing changes resulting from aggregation or reduced levels of nuclear TDP-43 may provide additional tools to assess the severity of TDP-43 dysfunction.

Several cryptic RNAs were found to accumulate in both AD-TDP and FTLD-TDP brains, in regions most affected by TDP-43 proteinopathy, but were absent from cognitively normal controls and AD no TDP control brains. In particular, we evaluated cryptic RNAs in *STMN2* and *UNC13A*, both of which we previously validated in FTLD-TDP cases [29, 35]. STMN2 is a neuronal phosphoprotein highly expressed during neuronal development, and in the adult brain [50]. It has been implicated in microtubule dynamics, has a role in axonal regeneration [51–53], and is important for maintenance of the neuromuscular junction [36–39]. While *STMN2* misprocessing due to TDP-43 loss of function has not been previously reported in AD, other stathmin protein family members have been implicated with AD pathogenesis. For example, STMN1 protein and RNA levels were decreased and upregulated, respectively, in AD brains [54]. Interestingly, the levels of STMN1 protein and number of NFT, but not plaques, were inversely correlated [54]. The accumulation of cryptic *STMN2* RNA in ALS/FTLD-TDP is accompanied by a decrease in full-length STMN2 RNA and protein [31, 32, 35]. Of interest, full-length STMN2 was found to be unaltered at both RNA and protein levels in AD; however, the TDP-43 status of these cases was not reported [55]. Thus, it is possible that cryptic *STMN2*, or the consequent decrease in full-length *STMN2* may play a pathogenic role in AD-TDP.

Genetic variants at the *UNC13A* locus were previously reported to confer an increased risk for ALS/FTD [56, 57], and recent studies demonstrate that the risk haplotype not only locates within the cryptic exon itself, but also leads to decreased ability of TDP-43 and other hnRNPs to bind and repress cryptic exon inclusion in *UNC13A* [29, 30, 58]. Of interest, reduced levels of Unc13a have been associated with impaired processing of amyloid precursor protein [59, 60], suggesting that changes affecting *UNC13A* splicing downstream of TDP-43 dysfunction may be detrimental in AD.

We also observed the accumulation of *CAMK2B* and *SYT7* cryptic RNAs in AD-TDP. These two genes encode proteins involved in calcium-dependent regulation of membrane trafficking and synaptic transmission. While less is known about whether these genes can influence AD, both have been associated with synaptic plasticity changes in mouse models of AD [61, 62]. Thus, our data further highlights the importance of changes in these genes to AD.

Dysfunction of TDP-43 in FTLD-TDP and AD-TDP also causes missplicing in the potassium channel regulator *KCNQ2*. In contrast to the other cryptic splicing events, loss of TDP-43 leads to the exclusion of an in-frame canonical exon in *KCNQ2*, rather than the inclusion of a novel exon. This “skiptic” event leads to an RNA encoding a protein lacking a sequence of the pore domain, which is required for potassium conduction. KCNQ2 forms part of M channels, and mutations in *KCNQ2* are associated with decreased neuronal excitability and neonatal epilepsy [63]. In fact, KCNQ2 has been associated with cognitive decline during normal aging [64], and M channel blockers have been proposed as potential therapeutic targets to treat cognitive decline in AD [63, 65]. Moreover, it has been suggested that amyloid beta deposition can affect KCNQ2 levels [66, 67], further supporting its potential contribution to AD pathophysiology.

The accumulation of cryptic RNAs paralleled the anatomic pattern of pTDP-43 accumulation, with high levels in both the amygdala and hippocampus of AD-TDP and FTLD-TDP. While the burden of cryptic RNAs initially seemed lower in AD-TDP cases compared to FTLD-TDP in both amygdala and hippocampus, these differences disappeared after correcting for age and sex. The accumulation of cryptic RNAs was also highly significant in the frontal cortex of FTLD-TDP, where the burden was similar to that in the amygdala, but AD-TDP frontal cortex showed no pTDP-43 deposition and no cryptic RNA accumulation. Given that the FTLD-TDP cases analyzed had a shorter disease duration (median 5 years, range: 1–25 years) than AD-TDP cases (median 10 years, range: 3–23 years), our results suggest that the accumulation of cryptic RNAs may be faster in FTLD-TDP. At the same time, deposition of TDP-43 pathology and cryptic RNAs in the frontal cortex in FTLD-TDP may explain a more aggressive disease phenotype in FTLD-TDP compared to AD-TDP. Why TDP-43 dysfunction in the frontal cortex is an early event in FTLD-TDP, but late in AD-TDP remains to be further studied.

We also evaluated whether different TDP-43 subtypes could influence the accumulation of TDP-43-regulated cryptic RNA targets. While some cryptic RNAs were present to a lower degree in FTLD-TDP type B and AD-TDP type β, there were not consistent expression patterns across targets or brain regions. This is intriguing for AD-TDP, since co-deposition of TDP-43 in NFT is common in AD-TDP type β, but it did not seem to affect TDP-43 dysfunction compared to AD-TDP type α. Overall, our studies suggest that cryptic RNA accumulation in both AD-TDP and FTLD-TDP results from loss of TDP-43 nuclear function, independently of TDP-43 inclusion type, and TDP-43 dysfunction in both AD and FTLD may result into shared disease mechanisms.

The specific contribution of cryptic RNAs to disease pathogenesis remains unclear. Inclusion of cryptic exons in some of these genes leads to premature stop codons (*STMN2*, *UNC13A*, *SYT7*, *CAMK2B*), which may lead to rapid degradation of the RNA or their respective truncated encoded proteins. Others result in the incorporation of in-frame exons without introducing stop codons (*KCNQ2*), and lead to the synthesis of stable proteins with potential deleterious functions. Indeed, some these predicted cryptic peptides (KCNQ2, CAMK2B, SYT7) have been identified in neurons depleted of TDP-43 and in the CSF of patients with ALS/FTD [34]. These studies not only underscore the importance of understanding the role of these de-novo proteins in disease pathogenesis, but also suggest that some may serve as markers of TDP-43 dysfunction. The ability to detect cryptic RNAs may differ due to transcript abundance, *KCNQ2* and *UNC13A* best associated with pTDP-43 burden in both AD-TDP and FTLD-TDP, and consistently differentiated TDP-43-positive cases from controls. Our observation that some of the same targets identified in AD-TDP as in ALS and FTLD-TDP suggests that biomarkers of TDP-43 dysfunction developed in ALS and FTD may serve to identify AD patients with TDP-43 pathology. Since TDP-43 pathology is associated with worse outcomes in AD [14–16], further understanding the role of missplicing events in AD is paramount.

Our study capitalized on a large cohort of clinically and pathologically well-characterized postmortem tissues, with analyses of three different brain regions relevant to TDP-43 deposition. In addition to inclusion of cognitively normal controls, cases of AD without TDP-43 pathology clearly aids in confirming that cryptic RNA accumulation in AD is dependent on TDP-43 pathology. Additional strengths of the study include use of high-quality tissues (high RIN) and our ability to quantify pTDP-43 and cryptic RNA burden. Our study also has some limitations. While we evaluated a few cryptic RNAs, additional cryptic RNAs have been described in ALS/FTLD-TDP which may also be misregulated in AD-TDP. One limitation of this study is that we do not know the specific contribution of these targets to disease. Thus, defining the functional impact of cryptic RNA accumulation to the pathobiology if AD is an essential future step. Another limitation is that only one method was used to determine the presence of cryptic RNAs. Additional methods such as analyses of RNA sequencing data or RNA-based imaging studies, while have their limitations, would be of added benefit as we have previously shown [29, 35]. Finally, while our data shows that *UNC13A* and *KCNQ2* were best able to discriminate TDP-43 cases from controls and they correlated better with pTDP-43 burden in both AD-TDP and FTLD-TDP, we cannot conclude which RNA or combination of RNAs may be the best candidate for biomarker development. In fact, measuring the levels of cryptic proteins may be more relevant to biomarker discovery efforts.

## Conclusions

Overall, we found that cryptic RNAs regulated by TDP-43 accumulate in tissues and diseases of different etiologies but with TDP-43 pathology. The fact that cryptic RNAs in ALS/FTLD-TDP also accumulate in AD-TDP cases suggest a common mechanism involving changes in RNA metabolism in both diseases. Our findings have significant implications for broadening our understanding of AD-TDP pathomechanisms and aiding in the development of biomarkers to identify AD cases with TDP-43 pathology. Future efforts to restore missplicing events resulting from TDP-43 dysfunction may be a promissing therapeutic approach for AD-TDP and ALS/FTLD-TDP. For example, restoring *STMN2* splicing is sufficient to restore axonal regeneration deficits resulting from TDP-43 dysfunction [32]. Further, the use of antisense oligonucleotides may serve as an effective tool to restore STMN2 splicing defects in vivo [42], and it is the goal of ongoing clinical trials (QRL-201 trial: NCT05633459). In all, TDP-43-regulated cryptic RNAs are expected to facilitate the generation of tools not only to assess TDP-43 dysfunction in ante-mortem biofluids, but also represent novel targets for therapeutic intervention, which would benefit multiple TDP-43 proteinopathies, including AD.

### Electronic supplementary material

Below is the link to the electronic supplementary material.

Supplementary Material 1

Supplementary Material 2

Supplementary Material 3

## Data Availability

All data generated or analyzed during this study are included in this published article [and its supplementary information files], or available from the corresponding author on reasonable request.

## Abbreviations

ALS: Amyotrophic lateral sclerosis
AD: Alzheimer’s disease
AD-TDP: AD with TDP-43 pathology
AUC: Area under the curve
CAMK2B: Calcium/calmodulin dependent protein kinase II beta
CI: Confidence interval
CN: Cognitively normal
CSF: Cerebrospinal fluid
FTLD: Frontotemporal lobar degeneration
FTLD-TDP: FTLD with TDP-43 pathology
KCNQ2: Potassium voltage-gated channel subfamily member 2
LATE: Limbic-predominant, age-related TDP-43 encephalopathy
NFT: Neurofibrillary tangle
RIN: RNA integrity number
ROC: Receiver operating characteristic
SEM: Standard error of the mean
STMN2: Stathmin 2
SYT7: Synaptotagmin 7
pTDP-43: Phosphorylated TDP-43
TDP-43: TAR DNA-binding protein 43 kDa
UNC13A: Unc-12 homolog A
