# Supplementary material for: TDP-43-regulated cryptic RNAs accumulate in Alzheimer’s disease brains
Source: Mol Neurodegener. 2023 Aug 21;18:57. doi: 10.1186/s13024-023-00646-z (PMC10441763; doi:10.1186/s13024-023-00646-z)
Supplement: Supplementary file 2 — Supplementary Material 2 [file 13024_2023_646_MOESM2_ESM.pdf]

Table S1. RNA integrity values for all samples in the study cohort. Related to Methods.

Study group	Amygdala	N	Hippocampus	N	Frontal cortex	N
Cognitively normal (CN)	9.3 (6.3, 10)	23	9.6 (6.0, 10)	27	9.6 (7.6, 10)	23
AD no TDP	9.7 (7.5, 10)	26	9.6 (7.7, 10)	27	9.3 (6.0, 10)	23
AD-TDP	9.3 (6.6, 10)	69	9.4 (5.9, 10)	71	9.2 (7.1, 10)	57
AD TDP type α	9.4 (7.0, 10)	34	9.4 (6.7, 10)	36	9.2 (7.1, 10)	26
AD TDP type β	9.3 (6.6., 10)	35	9.4 (5.9, 10)	35	9.2 (7.5, 9.9)	31
FTLD-TDP	9.2 (6.9, 10)	66	9.5 (6.7, 10)	67	9.6 (5.8, 10)	62
FTLD-TDP type A	9.2 (7.6, 9.9)	32	9.5 (6.7, 10)	32	9.4 (5.8, 10)	30
FTLD-TDP type B	9.2 (6.9, 10)	34	9.5 (7.4, 10)	35	9.7 (6.2, 10)	32

RNA integrity number (RIN) values are represented as median (max, min)

Table S2. Comparisons of insoluble pTDP-43 protein levels between AD-TDP and FTLD-TDP or control groups. Related to Fig. 1.

			Unadjusted analysis		Adjusting for age at death and sex	
Group	N	Median (minimum, maximum)	Regression coefficient (95% CI)	*P* value	Regression coefficient (95% CI)	*P* value
**Amygdala**						
CN	26	162.0 (141.5, 235.6)	-0.8895 (-1.0970 to -0.6821)	<0.0001	-0.8760 (-1.0900 to -0.6624)	<0.0001
AD no TDP	27	224.7 (176.5, 341.5)	-0.7737 (-0.9783 to -0.5690)	<0.0001	-0.7632 (-0.9773 to -0.5492)	<0.0001
AD-TDP	70	1021 (156.0, 15602)	0.00 (reference)	NA	0.00 (reference)	NA
FTLD-TDP	66	4759 (171.8, 15306)	0.4254 (0.2710 to 0.5798)	<0.0001	0.4284 (0.2454 to 0.6115)	<0.0001
**Hippocampus**						
CN	25	158.5 (120.0, 291.0)	-0.6018 (-0.7876 to -0.4160)	<0.0001	-0.5795 (-0.7715 to -0.3876)	<0.0001
AD no TDP	27	173.0 (136.5, 214.5)	-0.5842 (-0.7648 to -0.4035)	<0.0001	-0.5549 (-0.7443 to -0.3655)	<0.0001
AD-TDP	71	623.5 (129.5, 9393)	0.00 (reference)	NA	0.00 (reference)	NA
FTLD-TDP	67	1426 (165.0, 7094)	0.2619 (0.1529 to 0.3980)	0.0002	0.3070 (0.1450 to 0.4691)	0.0002
**Frontal cortex**						
CN	25	183.5 (160.5, 230.5)	-0.0692 (-0.2362 to 0.0978)	0.4145	-0.1267 (-0.2971 to 0.0438)	0.1443
AD no TDP	27	197.0 (173.5, 257.0)	-0.0308 (-0.1931 to 0.1316)	0.7089	-0.1005 (-0.2679 to 0.0670)	0.2379
AD-TDP	70	198.8 (165.0, 1457)	0.00 (reference)	NA	0.00 (reference)	NA
FTLD-TDP	67	6467 (189.0, 15145)	1.1800 (1.0580 to 1.3030)	<0.0001	1.0690 (0.9259 to 1.2120)	<0.0001

CN: cognitively normal controls; CI=confidence interval; β values, 95% CIs, and *P* values result from unadjusted linear regression models or linear regression models adjusted for age and sex. β values are interpreted as the difference in the mean levels of pTDP-43 between AD-TDP and the indicated groups. P values < 0.0167 are considered statistically significant after correcting for the comparisons of pTDP-43 protein between AD-TDP and 3 different groups.

Table S3. Comparisons of cryptic RNA levels between AD-TDP and FTLD-TDP or control groups in the frontal cortex. Related to Figure S1.

			Unadjusted analysis		Adjusting for age at death, sex, and RIN	
**Group**	**N**	**Median (minimum, maximum)**	**Regression coefficient (95% CI)**	***P* value**	**Regression coefficient (95% CI)**	***P* value**
STMN2						
Controls	46	0.012690 (0.001794, 0.084110)	-0.000515 (-0.005769 to 0.004740)	0.8469	-0.000915 (-0.006235 to 0.004404)	0.7344
AD-TDP	57	0.013720 (0.001118, 0.092850)	0.00 (reference)	NA	0.00 (reference)	NA
FTLD-TDP	62	0.040550 (0.001502, 0.260400)	0.014840 (0.009970 to 0.019700)	<0.0001	0.015070 (0.009473 to 0.020680)	<0.0001
KCNQ2						
Controls	46	0.000306 (0.00, 0.002998)	-0.000145 (-0.004060 to 0.003800)	0.9422	-0.001636 (-0.005811 to 0.002539)	0.4400
AD-TDP	57	8.65e-5 (0.00, 0.011430)	0.00 (reference)	NA	0.00 (reference)	NA
FTLD-TDP	62	0.018660 (0.00, 0.260200)	0.011790 (0.008135 to 0.015440)	<0.0001	0.008437 (0.004241 to 0.013030)	0.0002
UNC13A						
Controls	46	0.00 (0.00, 0.000771)	-7.56e-5 (-0.000912 to 0.000761)	0.8586	-0.000261 (-0.001127 to 0.000605)	0.5526
AD-TDP	57	0.00 (0.00, 0.006185)	0.00 (reference)	NA	0.00 (reference)	NA
FTLD-TDP	62	0.002430 (0.00, 0.031990)	0.002444 (0.001669 to 0.003219)	<0.0001	0.002092 (0.001180 to 0.003004)	<0.0001
CAMK2B						
Controls	46	0.000820 (0.00, 0.005377)	-8.49e-5 (-0.000554 to 0.000385)	0.7214	-0.000190 (-0.000681 to 0.000301)	0.4465
AD-TDP	57	0.000947 (0.00, 0.005477)	0.00 (reference)	NA	0.00 (reference)	NA
FTLD-TDP	62	0.002774 (0.000136, 0.017360)	0.001346 (0.000912 to 0.001781)	<0.0001	0.001107 (0.000590 to 0.001624)	<0.0001
SYT7						
Controls	46	0.000331 (0.00, 0.001927)	-5.07e-5 (-0.000624 to 0.000522)	0.8614	-0.000168 (-0.000776 to 0.000441)	0.5873
AD-TDP	57	0.000463 (0.00, 0.002081)	0.00 (reference)	NA	0.00 (reference)	NA
FTLD-TDP	62	0.003280 (0.00, 0.025390)	0.002020 (0.001489 to 0.002550)	<0.0001	0.001806 (0.001165 to 0.002446)	<0.0001

CI=confidence interval, RIN: RNA integrity number; regression coefficients, 95% CIs, and P values result from unadjusted linear regression models or linear regression models adjusted for age, sex and RIN. Regression coefficient values are interpreted as the difference in the mean levels of cryptic between AD-TDP and the indicated groups. P values < 0.025 are considered statistically significant after correcting for the comparisons of cryptic RNA between AD-TDP and 2 different groups.

Table S4. Comparisons of cryptic RNA levels between AD-TDP and FTLD-TDP or control groups in amygdala and hippocampus. Related to Fig. 2.

			Unadjusted analysis		Adjusting for age at death, sex, and RIN	
**Group**	**N**	**Median (minimum, maximum)**	**Regression coefficient (95% CI)**	***P* value**	**Regression coefficient (95% CI)**	***P* value**
**AMYGDALA**						
STMN2						
Controls	49	0.007544 (0.000337, 0.033620)	-0.006937 (-0.014130 to 0.000257)	0.0587	-0.009718 (-0.017120 to -0.002320)	0.0103
AD-TDP	69	0.015050 (0.00, 0.203300)	0.00 (reference)	NA	0.00 (reference)	NA
FTLD-TDP	66	0.041740 (0.002721, 0.379300)	0.015800 (0.009173 to 0.022430)	<0.0001	0.008414 (0.000813 to 0.016020)	0.0302
KCNQ2						
Controls	49	0.000209 (0.00, 0.002235)	-0.004677 (-0.008318 to -0.001030)	0.0122	-0.006240 (-0.010030 to -0.002447)	0.0014
AD-TDP	69	0.002810 (0.00, 0.103600)	0.00 (reference)	NA	0.00 (reference)	NA
FTLD-TDP	66	0.010920 (0.000183, 0.161900)	0.005252 (0.001893 to 0.008610)	0.0024	0.001691 (-0.002207 to 0.005589)	0.3931
UNC13A						
Controls	49	0.00 (0.00, 0.001030)	-0.000453 (-0.000968 to 6.18e-5)	0.0842	-0.000602 (-0.001140 to -6.37e-5)	0.0286
AD-TDP	69	0.000236 (0.00, 0.019850)	0.00 (reference)	NA	0.00 (reference)	NA
FTLD-TDP	66	0.000979 (0.00, 0.020540)	0.000758 (0.000283 to 0.001232)	0.0019	0.000344 (-0.000209 to 0.000897)	0.2217
CAMK2B						
Controls	49	0.000382 (0.00, 0.002236)	-0.000370 (-0.000729 to -9.87e-6)	0.0441	-0.000438 (-0.000816 to -5.92e-5)	0.0237
AD-TDP	69	0.000466 (0.00, 0.011240)	0.00 (reference)	NA	0.00 (reference)	NA
FTLD-TDP	66	0.001160 (0.00, 0.013630)	0.000468 (0.000135 to 0.000801)	0.0061	0.000237 (-0.000154 to 0.000627)	0.2329
SYT7						
Controls	49	0.000130 (0.00, 0.004001)	-0.000465 (-0.000882 to -4.88e-5)	0.0288	-0.000583 (-0.001024 to -0.000141)	0.0100
AD-TDP	69	0.000489 (0.00, 0.017920)	0.00 (reference)	NA	0.00 (reference)	NA
FTLD-TDP	66	0.001035 (0.00, 0.018650)	0.000311 (-7.28e-5 to 0.000695)	0.1116	6.45e-5 (-0.000389 to 0.000518)	0.7796
**HIPPOCAMPUS**						
STMN2						
Controls	54	0.007748 (0.001682, 0.033220)	-0.002226 (-0.004277 to -0.000176)	0.0335	-0.002984 (-0.005056 to -0.000911)	0.0050
AD-TDP	71	0.011220 (0.00, 0.058730)	0.00 (reference)	NA	0.00 (reference)	NA
FTLD-TDP	67	0.015180 (0.001127, 0.070690)	0.003295 (0.001371 to 0.005219)	0.0009	0.001931 (-0.000240 to 0.004103)	0.0810
KCNQ2						
Controls	54	0.000359 (0.00, 0.006249)	-0.045950 (-0.008079 to -0.001112)	0.0100	-0.005689 (-0.009402 to -0.001975)	0.0029
AD-TDP	71	0.002649 (0.00, 0.190400)	0.00 (reference)	NA	0.00 (reference)	NA
FTLD-TDP	67	0.006252 (0.00, 0.142000)	0.002268 (-0.001018 to 0.005553)	0.1750	0.000921 (-0.002996 to 0.004837)	0.6434
UNC13A						

Controls	54	0 (0.00, 0.000711)	-0.000234 (-0.000474 to 6.43e-6)	0.0564	-0.000313 (-0.000562 to -6.43e-5)	0.0139
AD-TDP	71	0.000157 (0.00, 0.004864)	0.00 (reference)	NA	0.00 (reference)	NA
FTLD-TDP	67	0.000694 (0.00, 0.01306)	0.000435 (0.000209 to 0.000662)	0.0002	0.000288 (2.57e-5 to 0.000551)	0.0316
CAMK2B						
Controls	54	0.000733 (0.00, 0.003402)	-0.000211 (-0.000465 to 4.30e-5)	0.1030	-0.000291 (-0.000559 to -2.40e-5)	0.0329
AD-TDP	71	0.000845 (0.00, 0.014710)	0.00 (reference)	NA	0.00 (reference)	NA
FTLD-TDP	67	0.001293 (0.00, 0.008700)	0.000183 (-5.67e-5 to 0.000422)	0.1338	6.84e-5 (-0.000214 to 0.000350)	0.6328
SYT7						
Controls	54	2.17e-11 (0.00, 6.98e-11)	-6.80e-12 (-1.25e-11 to -1.08e-12)	0.0202	-8.87e-12 (-1.49e-11 to -2.86e-12)	0.0040
AD-TDP	71	2.71e-11 (0.00, 2.02e-10)	0.00 (reference)	NA	0.00 (reference)	NA
FTLD-TDP	67	4.02e-11 (0.00, 2.84e-10)	5.14e-12 (-2.64e-13 to 1.05e-11)	0.0622	1.91e-12 (-4.43e-12 to 8.254e-12)	0.5527

CI=confidence interval, RIN: RNA integrity number; regression coefficents, 95% CIs, and *P* values result from unadjusted linear regression models or linear regression models adjusted for age, sex and RIN. Regression coefficient values are interpreted as the difference in the mean levels of cryptic between AD-TDP and the indicated groups. *P* values < 0.025 are considered statistically significant after correcting for the comparisons of cryptic RNA between AD-TDP and 2 different groups.

Table S5. Cryptic RNA accumulation associates with higher pTDP-43 protein levels in FTLD-TDP.

	Unadjusted analysis		Adjusting for age at death, sex, RIN and TDP-43 type	
**GROUP**	**Regression coefficient (95% CI)**	***P* value**	**Regression coefficient (95% CI)**	***P* value**
**Amygdala**				
STMN2	0.027210 (0.011900 to 0.042530)	0.0007	0.025760 (0.011450 to 0.040080)	0.0006
KCNQ2	0.011000 (0.003655 to 0.018540)	0.0041	0.010320 (0.003317 to 0.017320)	0.0046
UNC13A	0.001723 (0.000648 to 0.002798)	0.0021	0.001633 (0.000605 to 0.002661)	0.0023
CAMK2B	0.000853 (0.000131 to 0.001575)	0.0213	0.000786 (0.000120 to 0.001452)	0.0215
SYT7	0.000781 (4.06e-5 to 0.001520)	0.0390	0.000747 (1.29e-5 to 0.001482)	0.0462
**Hippocampus**				
STMN2	0.0005522 (-0.003578 to 0.004682)	0.7903	0.004492 (0.000354 to 0.008629)	0.0338
KCNQ2	0.009712 (0.003444 to 0.01598)	0.0029	0.009335 (0.002365 to 0.016310)	0.0095
UNC13A	0.0001355 (-0.0004544 to 0.0007254)	0.6480	0.000568 (-8.31e-5 to 0.001219)	0.0861
CAMK2B	-1.24e-5 (-0.0004359 to 0.0004111)	0.9535	0.000266 (-0.000211 to 0.000743)	0.4756
SYT7	1.157e-11 (1.123e-12 to 2.202e-11)	0.0305	9.51e-12 (-3.07e-12 to 2.21e-11)	0.1358
**Frontal cortex**				
STMN2	0.01373 (0.005827 to 0.02164)	0.0010	0.010120 (0.001074 to 0.019160)	0.0290
KCNQ2	0.01383 (0.00770 to 0.01997)	<0.0001	0.007644 (0.000855 to 0.014430)	0.0280
UNC13A	0.002583 (0.001240 to 0.003926)	0.0003	0.001986 (0.000541 to 0.003432)	0.0080
CAMK2B	0.001342 (0.0006186 to 0.002065)	0.0005	0.001086 (0.000261 to 0.001911)	0.0108
SYT7	0.002114 (0.001247 to 0.002982)	<0.0001	0.001979 (0.000959 to 0.002999)	0.0003

CI=confidence interval, RIN: RNA integrity number; regression coefficients, 95% CIs, and *P* values are shown for associations of cryptic RNA levels with pTDP-43 from unadjusted linear regression models or linear regression models adjusted for age, sex, RIN and TDP-43 subtype. *P* values < 0.01 are considered statistically significant.

Table S6. Association of cryptic RNA levels with TDP-43 subtype in FTLD-TDP.

		Unadjusted analysis		Adjusting for age at death, sex, RIN and pTDP-43 levels	
**Group**	**TDP-43 type**	**Regression coefficient (95% CI)**	***P* value**	**Regression coefficient (95% CI)**	***P* value**
**Amygdala**					
All RNAs	A	0.00 (reference)	NA	0.00 (reference)	NA
STMN2	B	0.007118 (-0.007004 to 0.021240)	0.3178	-0.003249 (-0.016090 to 0.009590)	0.6146
KCNQ2	B	0.000881 (-0.005864 to 0.007625)	0.7950	-0.004226 (-0.01500 to 0.002052)	0.1832
UNC13A	B	0.000497 (-0.000479 to 0.001474)	0.3126	-0.000158 (-0.001080 to 0.000764)	0.7329
CAMK2B	B	-0.000284 (-0.000919 to 0.000352)	0.3754	-0.000777 (-0.001374 to -0.000180)	0.0116
SYT7	B	0.000114 (-0.000536 to 0.000763)	0.7271	-0.000252 (-0.000911 to 0.000406)	0.4466
**Hippocampus**					
All RNAs	A	0.00 (reference)	NA	0.00 (reference)	NA
STMN2	B	0.001970 (-0.001634 to 0.005573)	0.2790	0.002314 (-0.001360 to 0.005987)	0.2127
KCNQ2	B	-0.005582 (-0.011330 to 0.000163)	0.0567	-0.005031 (-0.011200 to 0.001159)	0.1093
UNC13A	B	0.000139 (-0.000380 to 0.000658)	0.5941	0.000214 (-0.000365 to 0.000792)	0.4630
CAMK2B	B	0.000169 (-0.000202 to 0.000539)	0.3667	0.000152 (-0.000271 to 0.000575)	0.2694
SYT7	B	1.76e-13 (-9.36e-12 to 9.71e-12)	0.9707	1.22e-12 (-9.95e-12 to 1.24e-11)	0.8279
**Frontal cortex**					
All RNAs	A	0.00 (reference)	NA	0.00 (reference)	NA
STMN2	B	-0.013710 (-0.023350 to -0.004080)	0.0060	-0.009699 (-0.020860 to 0.001462)	0.0872
KCNQ2	B	-0.011760 (-0.019600 to -0.003919)	0.0039	-0.013310 (-0.021690 to -0.004933)	0.0024
UNC13A	B	-0.001774 (-0.003490 to -5.84e-5)	0.0429	-0.001061 (-0.002845 to 0.000723)	0.2385
CAMK2B	B	-0.000708 (-0.001640 to 0.000224)	0.1341	-0.000432 (-0.001450 to 0.000586)	0.3992
SYT7	B	-0.001103 (-0.002283 to 7.77e-5)	0.0666	-0.000305 (-0.001563 to 0.000954)	0.6298

CI=confidence interval, RIN: RNA integrity number; regression coefficients, 95% CIs, and *P* values are shown for associations of cryptic RNA levels with TDP-43 subtype from unadjusted linear regression models or linear regression models adjusted for age, sex, RIN and pTDP-43 levels. *P* values < 0.05 are considered statistically significant.

Table S7. Association of cryptic RNA levels with TDP-43 subtype in AD-TDP.

		Unadjusted analysis		Adjusting for age at death, sex, RIN and pTDP-43 levels	
**Group**	**TDP-43 type**	**Regression coefficient (95% CI)**	***P* value**	**Regression coefficient (95% CI)**	***P* value**
Amygdala					
All RNAs	α	0.00 (reference)	NA	0.00 (reference)	NA
STMN2	β	0.004484 (-0.002582 to 0.011550)	0.2096	0.007363 (3.93e-5 to 0.014690)	0.0488
KCNQ2	β	0.001649 (-0.000275 to 0.006040)	0.4564	0.003471 (-0.001190 to 0.008132)	0.1417
UNC13A	β	0.000288 (-0.000355 to 0.000794)	0.4476	0.000440 (-5.59e-5 to 0.000936)	0.1822
CAMK2B	β	0.000118 (-0.000337 to 0.000572)	0.6064	0.000306 (-0.000174 to 0.000785)	0.2071
SYT7	β	0.000132 (-0.000499 to 0.000763)	0.6776	0.000283 (-0.000419 to 0.000984)	0.4236
**Hippocampus**					
All RNAs	α	0.00 (reference)	NA	0.00 (reference)	NA
STMN2	β	-0.001099 (-0.003728 to 0.001530)	0.4072	-0.0002030 (-0.003208 to 0.002803)	0.8931
KCNQ2	β	-0.004504 (-0.009665 to 0.000656)	0.0861	-0.0004197 (-0.005887 to 0.005047)	0.8786
UNC13A	β	-7.95e-5 (-0.0002766 to 0.000117)	0.4420	5.687e-5 (-0.0001576 to 0.0002713)	0.5981
CAMK2B	β	-0.000135 (-0.000545 to 0.000275)	0.5131	-2.575-5 (-0.000496 to 0.0004445)	0.9132
SYT7	β	-3.86-12 (-1.20e-11 to 4.26e012)	0.3463	2.208e-13 (-8.374e-12 to 8.815e-12)	0.9562

CI=confidence interval, RIN: RNA integrity number; regression coefficients, 95% CIs, and *P* values are shown for associations of cryptic RNA levels with TDP-43 subtype from unadjusted linear regression models or linear regression models adjusted for age, sex, RIN and pTDP-43 levels. *P* values < 0.05 are considered statistically significant.
