# Supplementary material for: TDP-43-regulated cryptic RNAs accumulate in Alzheimer’s disease brains
Source: Mol Neurodegener. 2023 Aug 21;18:57. doi: 10.1186/s13024-023-00646-z (PMC10441763; doi:10.1186/s13024-023-00646-z)
Supplement: Supplementary file 3 — Supplementary Material 3 [file 13024_2023_646_MOESM3_ESM.pdf]

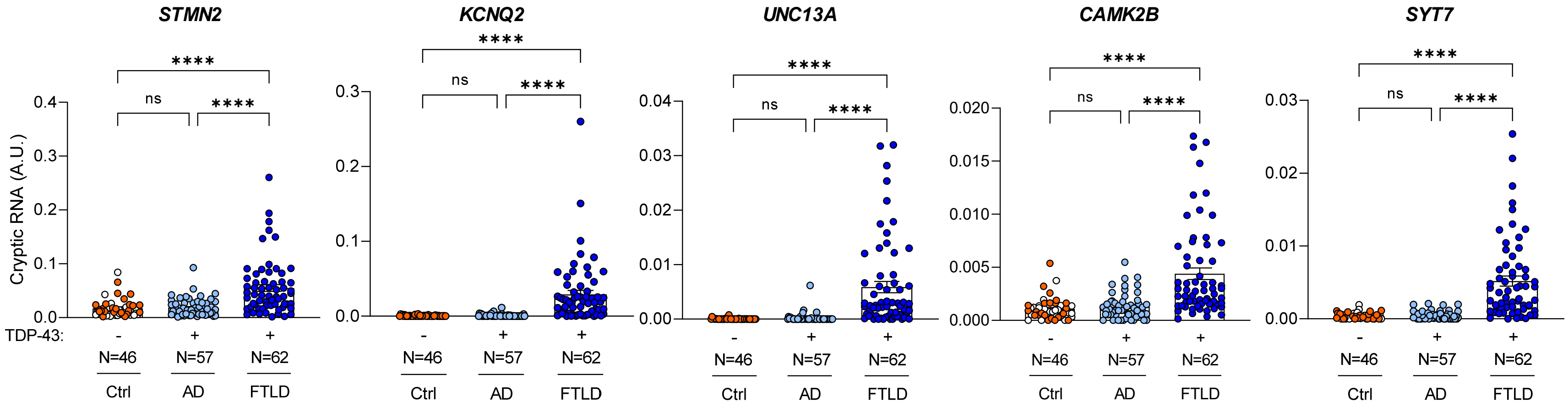

Figure S1. Cryptic RNAs accumulate in the frontal cortex of FTLD-TDP of our study cohort. Related to Fig. 2. Cryptic RNA (*STMN2, KCNQ2, UNC13A, CAMK2B, and SYT7*) levels were measured by qRT-PCR in the frontal cortex of controls (Ctrl; 23 CN, represented by white circles + 23 AD no TDP, represented by orange circles), 57 AD-TDP, and 62 FTLD-TDP cases. Data are presented as mean ± SEM. Statistical analyses were performed by One-way ANOVA following Dunn's multiple comparison tests: *P<0.05, **P<0.005, *** P<0.0005, ****P<0.0001, ns: not significant.

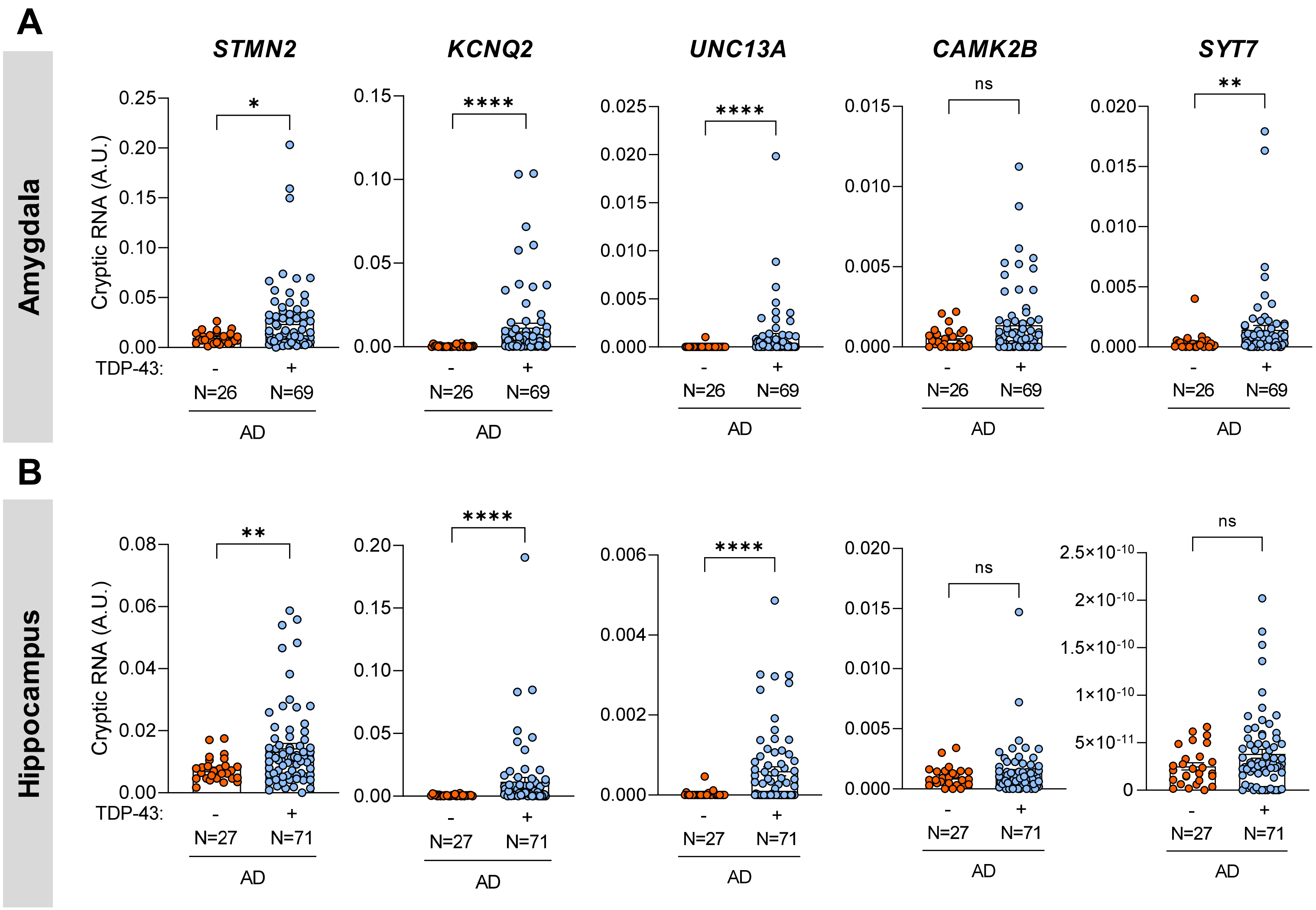

Figure S2. Cryptic RNAs accumulate in the amygdala and hippocampus of AD-TDP cases compared to AD no TDP. Related to Fig. 2. Cryptic RNA (*STMN2, KCNQ2, UNC13A, CAMK2B*, and *SYT7*) levels were measured by qRT-PCR in the amygdala (**A**) and hippocampus (**B**) of AD no TDP and AD-TDP cases. Number of cases is included in the figures. Data are presented as mean ± SEM. Statistical analyses were performed using the Mann-Whitney test: *P<0.05, **P<0.005, ****P<0.0001, ns: not significant.

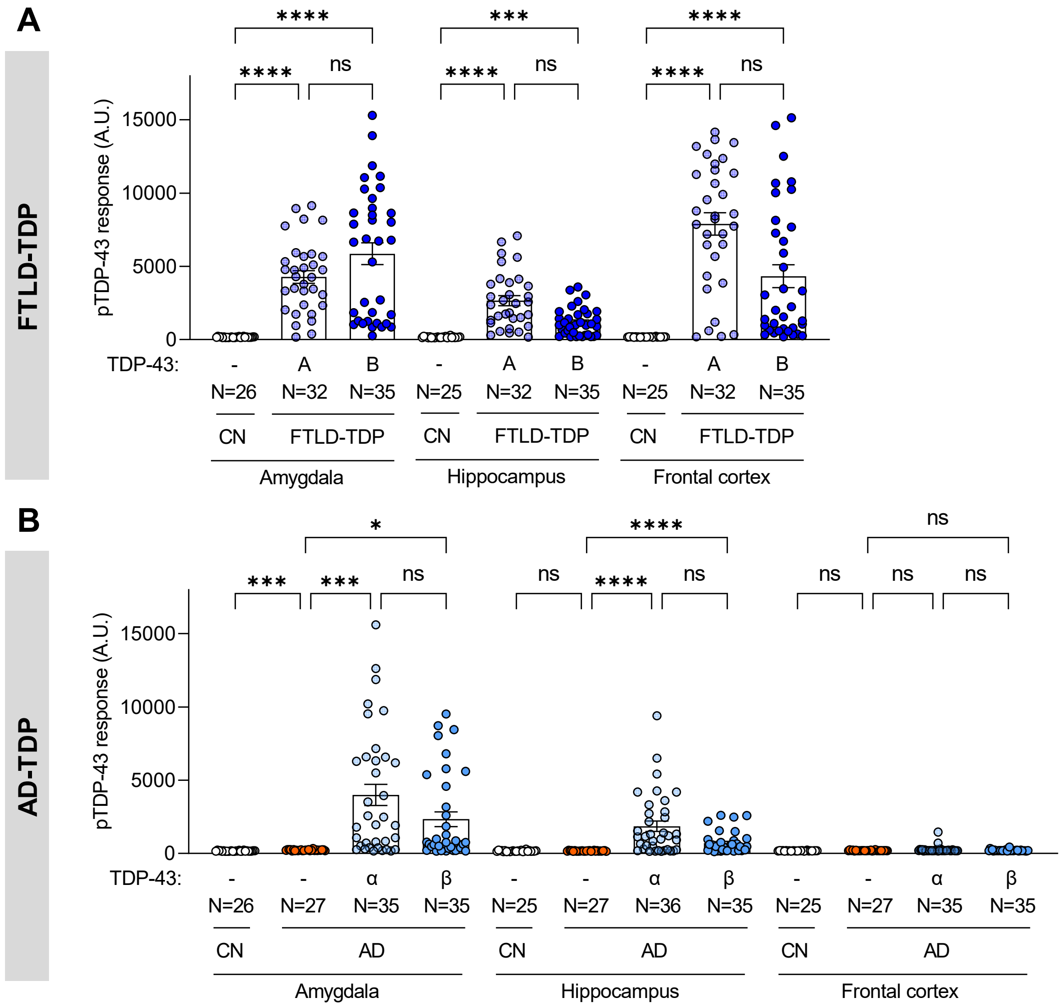

Figure S3. pTDP-43 burden in FTLD-TDP and AD-TDP by TDP-43 subtype. Related to Table 2. Quantification of pTDP-43 protein levels in FTLD-TDP (**A**) and AD-TDP (**B**), divided by TDP-43 subtype, and compared to cognitively normal controls (CN) and/or AD no TDP across three brain regions: amygdala, hippocampus, and frontal cortex (see **Table 1**), using an immunoassay (see **Methods**). Number of cases is included in the figures. Data are presented as mean ± SEM. Statistical analyses were performed by One-way ANOVA following Dunn's multiple comparison tests: *P<0.05, *** P<0.0005, ****P<0.0001, ns: not significant.

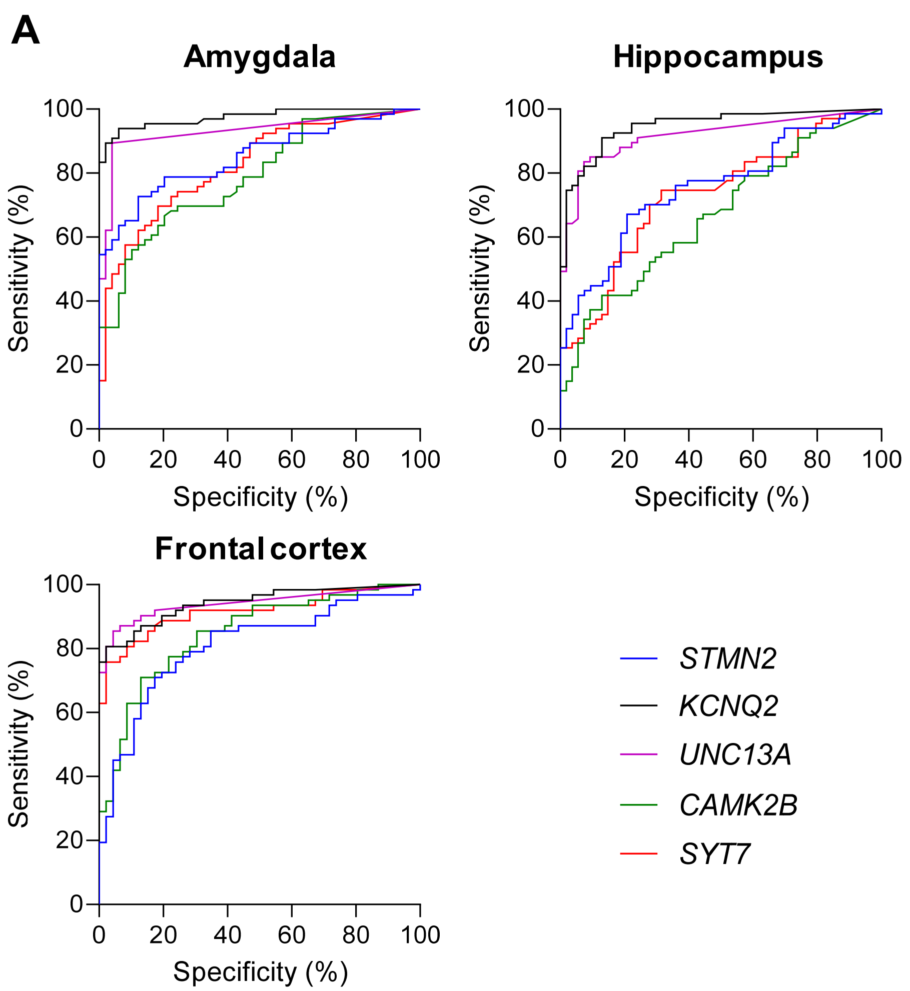

B

	AUC (95% CI)	p value
STMN2		
**Amygdala**	0.8506 (0.7821, 0.9192)	<0.0001
**Hippocampus**	0.7556 (0.6701, 0.8410)	<0.0001
**Frontal cortex**	0.8075 (0.7245, 0.8906)	<0.0001
KCNQ2		
**Amygdala**	0.9751 (0.9509, 0.9993)	<0.0001
**Hippocampus**	0.9469 (0.9087, 0.9852)	<0.0001
**Frontal cortex**	0.9423 (0.9005, 0.9841)	<0.0001
UNC13A		
**Amygdala**	0.9306 (0.8805, 0.9807)	<0.0001
**Hippocampus**	0.9168 (0.8652, 0.9684)	<0.0001
**Frontal cortex**	0.9411 (0.8951, 0.9871)	<0.0001
CAMK2B		
**Amygdala**	0.7879 (0.7068, 0.8690)	<0.0001
**Hippocampus**	0.6672 (0.5719, 0.7626)	0.0016
**Frontal cortex**	0.8475 (0.7749, 0.9201)	<0.0001
SYT7		
**Amygdala**	0.8231 (0.7479, 0.8983)	<0.0001
**Hippocampus**	0.7329 (0.6442, 0.8215)	<0.0001
**Frontal cortex**	0.9151 (0.8611, 0.9692)	<0.0001

Figure S4. Cryptic RNAs can discriminate FTLD-TDP cases from controls. Related to Fig. 3. (**A**) Representative images of the discriminatory ability of cryptic RNAs to distinguish FTLD-TDP cases from controls, evaluated by receiving operating characteristic (ROC) analyses, in amygdala (FTLD-TDP, N=66; controls, N=49), hippocampus (FTLD-TDP, N=67; controls, N=54) and frontal cortex (FTLD-TDP, N=62; controls, N=46). (**B**) The area under the curve (AUC) values, 95% confidence intervals (CI), and *P* values for each cryptic RNA are included for all cryptic RNAs in all brain regions.
